# Brief Exposure to a Self-Paced Computer-Based Reading Programme and How It Impacts Reading Ability and Behaviour Problems

**DOI:** 10.1371/journal.pone.0077867

**Published:** 2013-11-06

**Authors:** J. Antony Hughes, Gordon Phillips, Phil Reed

**Affiliations:** 1 Department of Psychology, Swansea University, Swansea, United Kingdom; 2 Glen Care Centre, Croydon, London, United Kingdom; University of Leicester, United Kingdom

## Abstract

Basic literacy skills underlie much future adult functioning, and are targeted in children through a variety of means. Children with reading problems were exposed either to a self-paced computer programme that focused on improving phonetic ability, or underwent a classroom-based reading intervention. Exposure was limited to 3 40-min sessions a week, for six weeks. The children were assessed in terms of their reading, spelling, and mathematics abilities, as well as for their externalising and internalising behaviour problems, before the programme commenced, and immediately after the programme terminated. Relative to the control group, the computer-programme improved reading by about seven months in boys (but not in girls), but had no impact on either spelling or mathematics. Children on the programme also demonstrated fewer externalising and internalising behaviour problems than the control group. The results suggest that brief exposure to a self-paced phonetic computer-teaching programme had some benefits for the sample.

## Introduction

Basic literacy levels are a concern in many countries. For example, the National Assessment of Adult Literacy in the USA found that 40% adults function in the lowest categories of ability [1]. In the UK, the reading ability of 20% of adults is below that expected of an 11 year old child [2]. Such poor literacy skills are also linked to poor future employment success [3], and to a worse prognosis for health [4]. Given such findings, literacy is seen by many governments as a key area to target for pupils in schools.

In fact, developing reading skills is paramount to the development of the pupil through school, and mastery of literacy skills will facilitate pupils' progress through the education system and, later, in the workplace. Individuals at risk from reading difficulties endure a slow and laboured progression to attain a similar level of academic achievement as their class peers [5]. Additionally, a struggling reader may have a greater tendency to exhibit problem behaviours [6], and have a tendency to display heightened signs of stress [7]. Thus, reading ability impacts both academic attainment, and behavioural and emotional problems [6]. An examination of internalising behaviours (e.g., anxiety, depression) and externalising behaviours (e.g., aggression, theft) in a sample of 6–8 year old children established a link between externalising behaviours and poor academic achievement, and also peer-victimisation. In turn, underachievement and peer-victimisation predicted greater internalising and externalising behaviours [8]. Similarly, first-grade pupils in the USA, who were identified as poor readers, displayed greater probabilities of poor task engagement, poor self-control, and externalising and internalising behaviour problems two years later [6]. Moreover, slow reading acquisition has been found to be associated with cognitive, behavioural, and motivational consequences; this prevents establishing optimum conditions for further cognitive development, as knowledge-based tasks that are dependent on reading skills are inhibited, attenuating development; in contrast, pupils with good reading skills advance at a rate where success generates further success, a phenomenon referred to as the ‘Matthew effect’ [9]. Thus, reading delay can be considered to have an impact upon the whole curriculum within schools.

Reading difficulties are linked to deficits in a variety of basic skills, such as: phonological processing [5], [10], sound discrimination [11], and letter-blending and segmenting [12], [13]. Furthermore, the ability to recognise words by sight at speed, requires practice and repetition, and a lack of such abilities will hinder reading skills [5], increasing the academic gap between pupils. To overcome some of these problems, the implementation of efficient phonics programmes has been suggested to ensure pupils become fluent readers [14]. A phonics programme, typically, begins with teaching very basic elements such as letter sounds, and proceeds to introduce sounds that are placed together and read at a quicker pace to produce ‘blending’. In this regard, pupils typically begin with constant-vowel-constant (CVC) words, such as ‘hen’, ‘rat’, ‘sun’, and later extend to more complex words [15]. Following this, pupils are introduced to digraphs, which are two sounds that together make one sound, such as ‘sh’, and ‘ch’.

Theoretically, phoneme-based programmes have suggested several routes to account for the success achieved, and it is considered that phonological abilities are a major cause of word reading skills [16]. For example, research assessing onset–rhyme skills and phoneme awareness as predictors of word recognition shows both phoneme sensitivity, and letter knowledge, to be predictors of subsequent word recognition [16]. As a consequence, interventions that incorporate phonemic awareness with letter knowledge serve to promote decoding skills in children with poor reading skills [17].

The beneficial effects of early implementation of a synthetic phonics and analytic programme have been demonstrated in a previous study [18]. The intervention reported in this study lasted 16 weeks, and included a multisensory approach to train synthetic phonics, via blending sounds together, for reading and spelling. Following training, participants illustrated an increase in reading ability seven months ahead of their chronological age. Similarly, intensive discrete phonic reinforcement and consolidation techniques have been studied [19]. In this programme, words were decoded, and then returned to their original forms (encoded), and children were encouraged to analyse and practice sound blends. There were improvements in reading accuracy, and comprehension, and 50% of the cohort achieved an increase of around two years in reading age within six months. Such structured phonics-based approaches are now considered more effective for the remediation of reading problems than non-phonetic methods [14], [20]. Recent phonic interventions employed in the UK include: ‘Catch Up Literacy’ [21], ‘Toe by Toe’ [22], ‘Sound Linkage’ [23], ‘Alpha to Omega’ [24], and ‘Literacy Acceleration’ [25].

However, a practical problem with many of these approaches is that they are one-to-one interventions, with a progressive developmental approach to teaching language skills, and the level of intervention required to improve reading ability is often quite intense, involving significant costs in terms of teacher time. One suggested solution to this problem involved the adoption of technologies to aid teachers cope with large classes of differing abilities [26]. Recent efforts in language intervention have utilised ICT, partly with the aim of reducing levels of direct teacher intervention, and making the programmes more accessible to greater numbers of children. However, success with such ICT-based interventions has been mixed [27]–[29]. In some of these studies, the relative lack of success has been attributed to a variety of factors such as a limited sample, that students were removed from normal lessons to take part in the intervention [27]. However, other research has demonstrated greater success. For example, the Finnish Remedial Reading Intervention (RRI) – a phonics programme taught in the classroom – and the Computer Assisted Remedial Reading Intervention (CARRI) – a combination of RRI and a graphogame that provides practice of letter-sound correspondence and phonemic awareness – provided good evidence of effectiveness in seven year-old school beginners in mainstream schools [29]. Such success points to the potential usefulness of computerised interventions to reinforce traditional methods of remedial interventions in mainstream schooling.

While this evidence supports the notion that computer programmes might be a significant advance and help in this area, there are clearly some issues to resolve in terms of documenting the effectiveness of these approaches and with regard to making such programmes more effective. One potential issue for such self-paced computer programmes is the degree to which they may motivate the children to participate through the appropriate delivery of reinforcers for their performance. Motivation to read has been identified as a major factor in developing this skill [30]. In the above studies, some studies report no use of reinforcers for pupil performance [27]. Others do report the use of particular reinforcers [28], [29], but do not report the degree to which these were viewed as effective by the pupils. This latter issue could be important for any self-paced computer-based reading intervention [31].

The current report aims to assess the effectiveness of the ‘Self Learn Read and Spell Programme’ in enhancing reading ability in secondary school aged pupils. This presents the stages of a phonics reading programme, via a self-paced computer-programme, in combination with performance-based access to pupil-chosen computer games. The computer games employed as reinforcers were developed in conjunction with software engineers, and had been pre-tested on pupils to gauge their engagement. After each stage of the phonic programme, the computer-programme allowed access to a computer-game (selected by the pupil), dependent upon the performance of the pupil in that previous stage. The primary aims of this research project were to add to the literature on the effectiveness of computer-assisted reading programmes relative to traditional approaches, and to explore the impact of brief exposure to the reading programme on changes in the pupils' reading ability, as previous programmes had been employed over considerable time periods. As a control to see the specificity of the intervention, the pupils' abilities in spelling and mathematics were also assessed, which the current reading programme might not be expected to impact so strongly, if at all. Additionally, the pupils' behavioural functioning, in terms of their externalising and internalising problems, was assessed to determine if exposure to the programme improved these aspects of behaviour.

## Method

### Ethics Statement

Ethical approval for this research was obtained from the Department of Psychology Ethics Committee, Swansea University. The participants and their next of kin/caretakers/guardians on their behalf provided their written consent to participate in this study. This procedure was approved by the Ethics Committee.

### Participants

Forty pupils (21 boys and 19 girls) attending an academy school in the south east of England were recruited for this study. The pupils were all from Year 7, and were all aged either 11 or 12 years. The pupils had all been identified as having reading problems by the school. The criteria for this were that, although the pupils had not been given a statement of special educational needs concerning dyslexia, that they were engaged in special reading classes at the school.

The mean IQ score (Wechsler Abbreviated Scale of Intelligence) for the sample was 83.7 (±12.8). The mean British Ability Scale (BAS) word reading ability age equivalent for the sample was 8∶11 (±1∶5) years, the mean spelling ability age equivalent was 8∶11 (±1∶4) years, and the mean maths ability age equivalent was 9∶9 (±1∶4) years. The sample was randomly divided into an intervention group (10 boys; 9 girls) and a control group (11 boys; 10 girls). The baseline characteristics of the four groups (boys and girls for each group) are shown in Table 1.

**Table 1:** Baseline characteristics of the groups in terms of mean (standard deviation) IQ (WASI full score), reading (BAS reading ability raw score), Spelling (BAS spelling ability raw score), maths (BAS maths ability raw score), externalising problems (SDQ total problems score), and internalising problems (CDI total T-score).

	Experimental		Control	
	Boys	Girls	Boys	Girls
WASI	85.5 (11.1)	82.0 (8.6)	87.0 (18.)	80.0 (11.0)
BAS Read Raw	32.0 (10.1)	37.9 (6.7)	35.0 (14.9)	36.9 (14.1)
BAS Spell Raw	12.5 (6.7)	18.7 (5.1)	14.3 (9.7)	17.3 (11.4)
BAS Maths Raw	10.9 (3.5)	11.1 (4.2)	11.3 (4.0)	8.9 (4.0)
SDQ Total	8.0 (3.8)	5.3 (3.4)	9.9 (5.5)	5.3 (1.4)
CDI Total (T-score)	49.2 (8.7)	56.1 (9.8)	49.0 (5.9)	49.0 (9.8)

These baseline data were all subject to two-factor between-subject analysis of variance (ANOVA), with intervention (experimental versus control) and gender (boy versus girl) as factors. There were no di fferences for reading ability (BAS raw score): gender, *F*<1, intervention, *F*<1, interaction, *F*<1; spelling ability (BAS raw score): gender, *F*(1,36) = 3.27, *p*>.07, intervention, *F*<1, interaction, *F*<1; maths ability (BAS raw score): gender, *F*<1, intervention, *F*<1, interaction, *F*<1; or internalising problems (Children's Depression Inventory; CDI Total T-Score): gender, *F*(1,36) = 1.76, *p*>.10, intervention, *F*(1,36) = 1.97, *p*>.10, interaction, *F*(1,36) = 1.76, *p*>.10. The groups did differ in terms of their externalising behaviour problems (Strengths and Difficulties Questionnaire; SDQ total problems score), where the ANOVA revealed a statistically significant main effect of gender, *F*(1,36) = 6.67, *p*<0.05, the boys displaying greater externalising problems than the girls, but there was no statistically significant main effect of intervention nor interaction between the factors both *F*s<1. Thus, the intervention groups were well matched at baseline.

### Measures

***Wechsler Abbreviated Scale of Intelligence*** (WASI, 31) measures intellectual ability, and is suitable for ages 6 to 89 years. It comprises four subtests, two assessing language (vocabulary and similarities), and two performance measures (block design and matrix reasoning). Thus, the WASI generates two scores of abilities, verbal and performance scores, and a full score of intellectual functioning. Test reliability has been stated at .87 to .92.

***British Abilities Scale*** (BAS II, 32) includes three achievement scales (reading, spelling, and maths), which index educational achievement. It is suitable for use with children and adolescents from two years, six months old (2∶6) to seventeen years, eleven months old (17∶11) [32]. During the development of the scales, good internal and test-retest reliability were observed, and internal reliability of the achievement scales for the age range of 11 years (11.0) to eleven years eleven months (11.11) has been stated as .91 (maths), .93 (spelling) and .95 [33]. The scales also correlated well with the Wechsler Intelligence Scale for Children - Third Edition [34].

***Strengths and Difficulties Questionnaire*** (SDQ, 33) is a brief behavioural screening questionnaire concerning 3 to 16 year olds. It exists in several versions to meet the needs of researchers, clinicians, and educationalists. The teacher version includes 25 items, divided between 5 scales, all score ranges 0 to 10: emotional symptoms (norm = 1.9), conduct problems (norm = 1.6), hyperactivity/inattention (norm = 3.5), peer relationship problems (norm = 1.5), and pro-social behaviour. The first four sub-scales, when added together, generate a Total Difficulties Score (norm = 8.4; score range 0–40). The internal reliability of the scales ranges from 0.69 to 0.85 [36]. Recent tests of validity have demonstrated consistency with the Rutter questionnaires, whilst offering a positive behavioural measure [35]; differentiation between child psychiatric samples and community samples [37].

***Children's Depression Inventory 2^nd^ Edition*** (CDI II, 37) assesses the severity of teacher observed symptoms of childhood depression in children between the ages of 7–17 years, and can be administered by reading to those with poor reading skills. It produced an overall depression score (T-score). The internal reliability of the scale is 0.86, and convergent validity of the CDI II with Conner's Comprehensive Behavior Rating Scales, and the Beck Depression Inventory – Youth Version, provided positive and significant correlations, whilst also providing discrimination [38].

### Intervention

**Figure 1:**
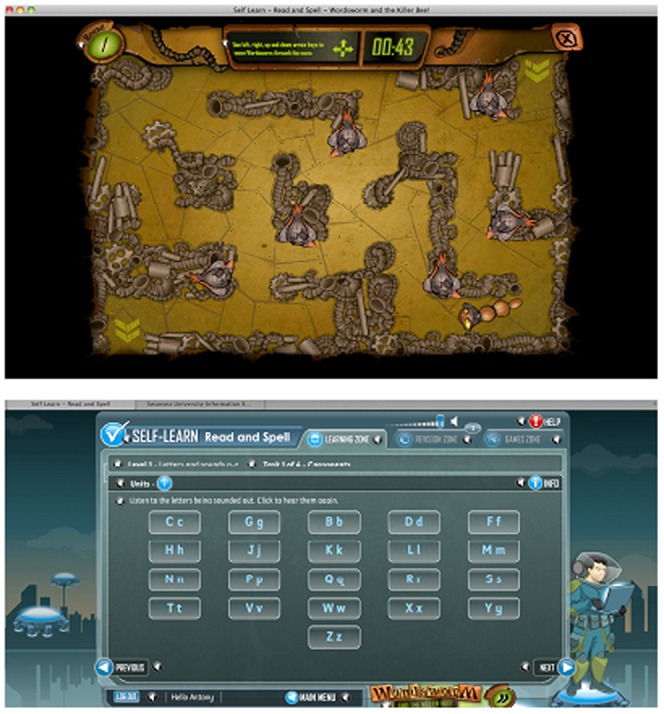
Screen shots of computer game used as a reinforcer (top panel), and part of the teaching programme (bottom panel).

The ‘self-learn read and spell’ computer programme has been developed for individuals with reading problems, and has been in use for several years. It is based around an attempt to improve phonetic discrimination. The programme has been designed for 8 to 20 year olds, which is reflected in the four computer games built into the programme that serve as reinforcers for completing each stage of teaching (‘Dinnertime for Deep Sea Dora’, ‘Escape from Planet Lexicon’, ‘Billy Bob's Rifle Range’, and ‘Sir Spellalot's Big Adventure’). All of the games have phonics features to help practice with the skills learned in the programme. These computer games were developed in traditional iterative manners for such games involving initial development, testing on the target age group, and then refinement. A screen shot of one of the games is seen in the top panel of Figure 1.

The programme has 23 levels of teaching. For example, it starts with a review of the alphabet (Level 1), and then moves to a basic phonic word teaching phase (Level 2). Three-letter phonic words are contained in Level 2 (e.g., cat, kit, fun, jet, pan, tap, etc). This gives the user practise in the constant combinations, vowel blending, and phonic combination, with easy words, which will give them confidence and the ability to spell 150 words. Level 3 moves onto four-letter words, with a combination of two letters with a single and double sound (e.g., th, wh, ng, sh, ch, qu, ck). Levels 5 and 6 cover the silent ‘e’ (fat–fate), and Level 7 deals with double vowels where only one vowel is sounded (e.g., boat, dead, steak). Level 8 teaches the ‘o’ sounds (e.g., hop, hope, cold, cow, rough), and Level 9 highlights the ‘oo’ sound (e.g., boot, blue, crew). A screen shot of one of the teaching levels is shown in the bottom panel of Figure 1, and all of the levels and stages are shown in Table 2.

**Table 2:** Levels and tasks in the Self Learn Read and Spell Programme.

Level 1: letters and sounds a – z	Task 1 – consonants
	Task 2 – Vowels
	Task 3 – Other sounds
Level 2: three letter phonic	Task 1 – Quick revision
words	Task 2 – at
	Task 3 – ‘o’ in the middle
	Task 4 – ‘e’ ‘i’ and ‘u’ in the middle
	Task 5 – Everyday words that you should know
	Task 6 – Reading practice
Level 3: four letter phonic words	Task 1 – Quick revision
	Task 2 – phonic sounds at the start
	Task 3 – special sounds at the start
	Task 4 – special sounds at the end
	Task 5 – Everyday words that you should know
	Task 6 – Reading practice
Level 4: four and five letter	Task 1 – Quick revision
phonic words – endings	Task 2 – ‘ng’ sounds at the end
	Task 3 – ‘ll’ sounds at the end
	Task 4 – ‘oo’ and ‘sh’ sounds at the end
	Task 5 – ‘ck’ sounds at the end
	Task 6 – other sounds at the end
	Task 7 – Everyday words that you should know
	Task 8 – Reading practice
Level 5: Long vowels and short	Task 1– Quick revision
vowels	Task 2 – Adding the magic ‘e’
	Task 3 – Everyday words that you should know
	Task 4 – Reading practice
Level 6: Magic ‘e’ words	Task 1 – Quick revision
	Task 2 – ‘a’ long
	Task 3 – ‘i’ long
	Task 4 – ‘o’ long and ‘u’ long
	Task 5 – Funny combinations with a vowel and ‘e’ at the end
	Task 6 – Everyday words that you should know
	Task 7 – Reading practice
Level 7 – two vowels together	Task 1 – Quick revision
	Task 2 – first vowel long
	Task 3 – second vowel long
	Task 4 – unique double vowel sounds
	Task 5 – ear - same letters but different sounds!
	Task 6 – oo - same letters but different sounds!
	Task 7 – ou - same letters but different sounds!
	Task 8 – Everyday words that you should know
	Task 9 – Reading practice
Level 8: the trouble with ‘o’	Task 1 – Quick revision
	Task 2 – ‘o’ can follow the short vowel rule
	Task 3 – ‘o’ can follow the long vowel rule (add the ‘magic e’)
	Task 4 – ‘o’ can follow the ‘first vowel long’ rule
	Task 5 – ‘o’ can sound ‘o’
	Task 6 – ‘o’ can sound ‘ow’
	Task 7 – ‘o’ can sound ‘uh’
	Task 8 – ‘o’ can sound ‘or’
	Task 9 – ‘o’ can sound ‘oi’
	Task 10 – ‘o’ can sound ‘oo’
	Task 11 – ‘o’ can sound ‘oo’
	Task 12 – ‘o’ can sound ‘w’
	Task 13 – Everyday words that you should know
	Task 14 – Reading practice
Level 9: Sounds like ‘oo’ and ‘o’	Task 1 – Quick revision
and ‘ow’ and ‘ee’	Task 2 – sounds like ‘oo’
	Task 3 – sounds like ‘o’
	Task 4 – sounds like ‘ow’
	Task 5 – sounds like ‘ee’
	Task 6 – Everyday words that you should know
	Task 7– Reading practice
Level 10 : The ‘u’ and ‘ough’	Task 1 – Quick revision
	Task 3 – how many ways can you say ‘ough’?
	Task 4 – Everyday words that you should know
	Task 5– Reading practice
Level 11: Sounds like ‘or’ and ‘er’	Task 1 – Quick ‘A’ revision
	Task 2 – sounds like ‘or’
	Task 3 – sounds like ‘er’
	Task 4 – sounds like ‘a’
	Task 5 – Everyday words that you should know
	Task 6 – Reading practice
Level 12: Sounds like ‘i’ and ‘u’	Task 1 – Quick revision
and ‘oi’ and ‘r’	Task 2– the long ‘i’ sound
	Task 3 – the long ‘u’ sound
	Task 4 – sounds like ‘oi’
	Task 5 – sounds like ‘r’
	Task 6 – Everyday words that you should know
	Task 7– Reading practice
Level 13: Sounds you don’t	Task 1 – Quick revision
expect!	Task 2 – sounds like ‘ser’
	Task 3 – sounds like ‘c’
	Task 4 – sounds like ‘f’
	Task 5 – sounds like ‘s’
	Task 6 – sounds like ‘w’
	Task 7 – Everyday words that you should know
	Task 8 – Reading practice
Level 14: Adding to words at the	Task 1 – Quick revision
end	Task 2 - ending with single consonant
	Task 3 words ending with a double consonant
	Task 4 – Words ending in h, k, r, x, w and y
	Task 5 – Words with a long vowel
	Task 6 – Everyday words that you should know
	Task 7 – Reading practice
Level 15: Adding to words at the	Task 1 – Quick revision
end – ‘ive’, ‘ous’ and words that	Task 2 – adding ‘ive’
end in ‘y’	Task 3 – adding ‘ous’
	Task 4 – adding to words that end in ‘y’
	Task 5 – Everyday words that you should know
	Task 6– Reading practice
Level 16: Adding to words at the	Task 1 – Quick revision
end – ‘ial’ and ‘ion’	Task 2 – adding ‘ial’
	Task 3 – adding ‘ion’
	Task 4– Everyday words that you should know
	Task 5 – Reading practice
Level 17: Adding to words at the	Task 1 – Quick revision
end - ‘ic’, ‘able’, ‘ible’, ‘ly’, ‘ment’	Task 2 – Adding ‘ic’
and ‘less’	Task 3 – Adding ‘ible’ and ‘able’
	Task 4 – Adding ‘ly’
	Task 5 – Adding ‘ment’
	Task 6 – Adding ‘less’
	Task 7 – Everyday words that you should know
	Task 8 – Reading practice
Level 18: Adding to words at the	Task 1 – Quick revision
beginning	Task 2 – adding ‘un’
	Task 3 – adding ‘re’
	Task 4 – adding ‘im’
	Task 5 – adding ‘dis’
	Task 6 – adding ‘ir’
	Task 7 – adding ‘in’
	Task 8 – Everyday words that you should know
	Task 9 – Reading practice
Level 19 – Words with a silent	Task 1 – Quick revision
	Task 2 – silent letters
	Task 3 – Everyday words that you should know
letter	Task 4 – Reading practice
Level 20 – ‘i' before ‘e’ except	Task 1 – Quick revision
after ‘c’	Task 2 – ‘i’ before ‘e’ except after ‘c’
	Task 3 – ‘e’ before ‘i’
	Task 4 – Everyday words that you should know
	Task 5 – Reading practice
Level 21 – Homophones	Task 1 – Quick revision
	Task 2 – there their they’re
	Task 3 – hear here
	Task 4 – Everyday words that you should know
	Task 5 – Reading practice
Level 22 – Capital letters	Task 1 – Quick revision
	Task 2 – names of people
	Task 3 – names of places
	Task 4 – days of the week
	Task 5 – months of the year
	Task 6 – Everyday words that you should know
	Task 7 – Reading practice
Level 23 – PLURALS	Task 1 – Quick revision
	Task 2 – Adding ‘s’
	Task 3 – Adding ‘es’ and ‘ies’
	Task 4 –‘ife’ and ‘f’ at the end
	Task 5 – Adding ‘s’ and ‘es’ to words that end in ‘o’
	Task 6 – the odd ones!
	Task 7 – Everyday words that you should know
	Task 8 – Reading practice

Pupils must score 80% on the test before the move onto the next level, and any incorrect words are placed in a revision folder to learn on the next level. The folder has a red, amber, and green ‘traffic light’ system, whereby unrecognised words are represented as red, indicating that they need to be learned, but once this has been achieved the words moves to amber, and, if after a further test proves positive, they turn green. However, should the user again fail the test, the words will remain amber until such time as they are correct.

Successful completion of each level of the programme allows access to one of the computer games. At all levels, the pupil can go into the games zone for a specified period depending on their performance in the test in that level. If they have scored 80% in the tests, they got 5 min of time in the game zone; if 90% was scored in the tests, they got 10 min; and, when 95% was scored, they got 15 min.

### Procedure

The pupils were tested, in the school, by an appropriately trained psychologist (who was also a qualified teacher), and who administered the WASI, and the BAS achievement scales. At the same time, the psychologist asked the pupil's teacher to complete the SDQ and CDI about the children selected for inclusion. The children were then randomly divided into two groups: one group completed six weeks of exposure to the above computer programme; and the other group remained in their normal reading classes for that period of time. These reading classes comprised the use of eclectic methods for teaching reading, including phonics and whole-word techniques, but were not delivered by a computer. Both groups of children experienced three, 40 min sessions a week of their intervention, for a period of six weeks. In both groups, the children worked in small groups (4–6) in a class with a teacher and a learning support assistant. In the case of the computer programme, the children worked singly at a computer at there own pace through the programme, and could ask for assistance if they needed help. After the six weeks, the pupils were reassessed by the same psychologist in terms of their BAS scores, and the same teacher again completed the SDQ and CDI.

## Results

**Figure 2:**
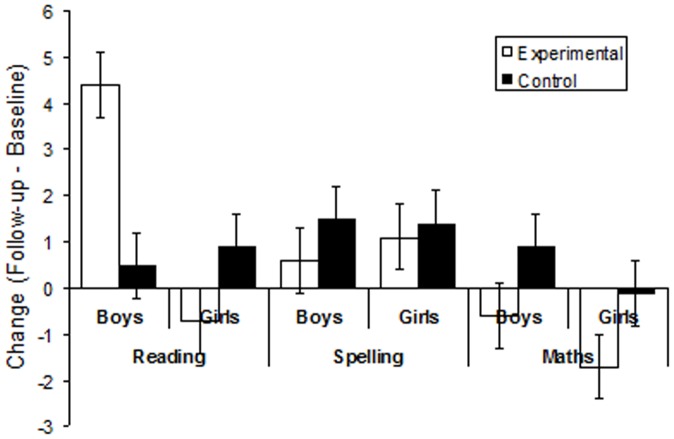
Mean in change (follow-up minus baseline) in BAS raw scores for the two groups (experimental  =  computer-based programme; control  =  normal reading classes) for both boys and girls.

Figure 2 shows the change in the raw scores (follow-up minus baseline) for the three BAS achievement scales for all groups. Using the change scores is a widely adopted procedure in the literature relating to the effectiveness of interventions, and also accommodates differences in baseline measures without having to include those measures in an analysis of covariance, which would involve assumptions about the manner in which baseline and outcome scores were related, and dramatically increases the needed sample size, thus, reducing power to detect change. Inspection of these data reveals that the boys exposed to the programme demonstrated relatively larger gains than the boys not exposed to the programme, but that there was very little difference between the two groups of girls. There was little difference between the change scores of the groups for either spelling or maths ability

These impressions were corroborated by separate two-factor between-subject analyses of variance (ANOVA) conducted on the reading, spelling, and maths change scores, with intervention (experimental versus control) and gender (boys versus girls) as factors. The ANOVA conducted on the reading change score revealed no significant main effect of intervention, *p*>.20, but a statistically significant main effect of gender, *F*(1,36) = 5.89, *p*<0.05, and a statistically significant interaction between the two factors, *F*(1,36) = 7.75, *p*<.01. Simple effect analyses conducted on the boys and girls separately revealed a statistically significant simple effect of intervention for the boys, *F*(1,36) = 7.93, *p*<.01, but not for the girls, *F*<1. The ANOVA conducted on the change in spelling scores revealed no statistically significant main effects or interactions, all *p*s>.40. Similarly, the ANOVA conducted on the change in maths scores revealed no statistically significant main effects or interactions, all *p*s>.05.

**Figure 3:**
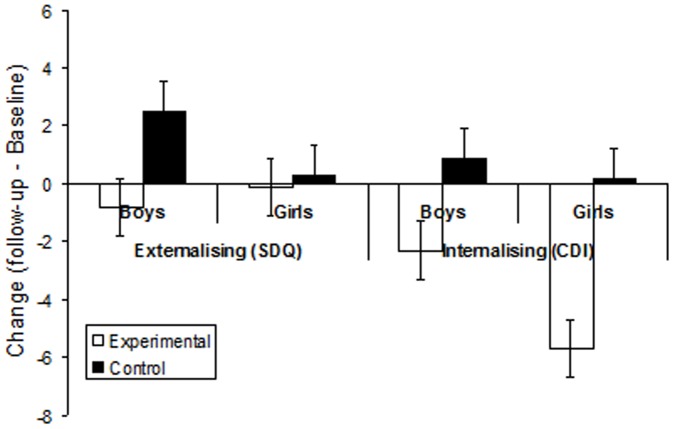
Mean in change (follow-up minus baseline) in externalising (SDQ) and internalising (CDI) scores for the two groups (experimental  =  computer-based programme; control  =  normal reading classes) for both boys and girls.

Figure 3 shows the change in the scores (follow-up minus baseline) for the externalising behaviours (total SDQ problem score), and internalising problems (CDI T-score) for all groups. Inspection of these data reveals that the pupils exposed to the programme demonstrated relative decreases in their internalising and externalising problems compared to pupils not exposed to the computer programme. In the case of the externalising problems, this relative improvement was largely the product of the externalising problems not getting worse in the computer-group compared to them deteriorating more in the control group. These impressions were corroborated by separate two-factor between-subject ANOVAs conducted on the externalising (SDQ) and internalising (CDI) problems, with intervention (experimental versus control) and gender (boys versus girls) as factors. The ANOVA conducted on the externalising change score revealed a statistically significant main effect of intervention, *F*(1,36) = 3.75, *p*<.05, but no main effect of gender, or interaction, both *p*s>.20. The ANOVA conducted on the change in internalising problems revealed a statistically significant main effect of intervention, *F*(1,36) = 5.80, *p*<.05, but no main effect of gender, or interaction, both *p*s>.20.

## Discussion

The current study assessed the impact of a self-paced computer programme designed to teach phonics on the academic and behavioural functioning of secondary-aged pupils. The results demonstrated that brief exposure to the programme (six weeks) led to improvements in reading for boys (but not girls), and during the period of intervention, the behaviour problems in both boys and girls exposed to the programme improved relative to a control group receiving additional tuition in reading but who were not using the computer programme.

The fact that reading ability was improved by exposure to a computer-based reading programme suggests that such self-paced computer-approaches may have utility in developing reading abilities at a greater rate. These impacts on reading suggest that the improvements noted with other computer-programmes [29], employed over longer periods of time, can be replicated, even with relatively short exposures. That exposure to the current computer programme did improve reading, while several other demonstrations showed little improvement [27], [28], may potentially be attributable to a variety of factors, which will need exploration. However, the employment of effective reinforcers in the current programme, which was integral to development of the computer-programme, may be of note [26]. Certainly, the use of appropriately selected reinforcers has been shown to be a necessary component in reading programmes [31]. Although some consideration of potential impacts of such reinforcement-based schemes on pupil's intrinsic motivation is required [39], a longer follow-up period would be needed to establish this, and it should be noted that there are many demonstrations of reinforcement having no long-term detrimental effect on pupil's motivation to read [40].

The relative benefits of the computer-programme in helping both externalising- and internalising-behaviour problems were also encouraging, and replicate a previous demonstration of this effect [6]. However, while there were improvements in the behaviours of the pupils exposed to the computer programme, this effect seems mainly to be the product of the behaviours of the children in the comparison groups worsening. It is unclear why this would be the case, it might be the case that this is the normal course of development for such a sample, or that their relative inability to read produced greater numbers of behavioural problems. This latter suggests maps onto a further question that remains concerning these findings, which is whether the impacts on reading and behaviour are independent, or mediate, one another; that is, does improvement in reading lead to improvements in behaviour, or vice versa? With a short intervention, and relatively few participants, this is a difficult question to explore with the current data. However, a preliminary mediation-analysis performed on these data for the boys, showed no suggestion that either behaviour mediated the effect of the intervention on reading, or vice versa. The point biserial correlations between intervention and reading outcome, or intervention and behavioural outcomes, were not impacted by the addition of a mediator.

The difference in the impact of the computer-programme on the reading abilities of boys and girls is a finding that needs further exploration. On the one hand, it may suggest that the specific reinforcers built into the programme may have been more effective for boys compared to girls; and on the other hand, it may suggest that the computer-based approach, as a whole, may be more suited to boys than girls. However, these suggestions need to be tempered by the finding that exposure to the programme did impact on the behaviour problems exhibited by both the girls and the boys, suggesting that some aspects of the intervention were effective for girls. It also could be noted that the girls had a marginally (but not statistically significantly) higher reading ability at the start of the programme than the boys, and this might point to the intervention being more effective with poorer readers. However, this consideration needs to be tempered by the fact that all of the participants were actually poor readers, and these boy-girl differences were marginal. Moreover, further analysis of these reading outcome data, using baseline reading ability as a covariate, had no impact on the pattern of statistical significance reported above, suggesting baseline reading ability was not a contributory factor.

There are a number of limitations to the study, which should be acknowledged. The measures of reading were limited to the BAS achievement scales, which do not reflect fluency or comprehension, and these aspects of reading will need further exploration. The intervention was also very brief, and, while this is encouraging in one respect, the impact of longer interventions will need to be explored. Moreover, the gains in reading will need to be studied for their maintenance after programme termination. In addition, future work might include a comparison of the current programme with other phonetic computer-teaching programmes [41]. Finally, it should be noted that the current sample did not have defined reading problems, but were identified by the school as having such problems, which may mean they were poor-performing in the context of that school, but might not have ‘diagnosable’ reading difficulties. This means that further work will be needed to see if the current results generalize to such a specific sample.

In terms of the programme, there are still some developments that could be suggested to improve its effectiveness. The games used as reinforcers could be subject to a stronger reinforcer assessment test, to determine their effectiveness in this capacity. A reinforcer is not determined, as such, by its nature, but by its effects on the frequency of that successful performance in the future, and while the games have been identified as acceptable and pleasurable by the children, the introduction of reinforcer assessment tests, and some aspects of child choice into the programme may facilitate this aspect of the programme.

In summary, the current report demonstrated that brief-exposure to a computer-based reading programme can have an impact on reading abilities, and also impacts positively on both externalising-behaviours and internalising-behaviour problems. This adds to the literature indicating that such approaches may well be effective, and a potentially useful addition to the set of tools available to teachers.
